# Supplementary figures and images for: Podocyte-specific knockout of the neonatal Fc receptor (FcRn) results in differential protection depending on the model of glomerulonephritis
Source: PLoS One. 2020 Dec 28;15(12):e0230401. doi: 10.1371/journal.pone.0230401 (PMC7769425; doi:10.1371/journal.pone.0230401)

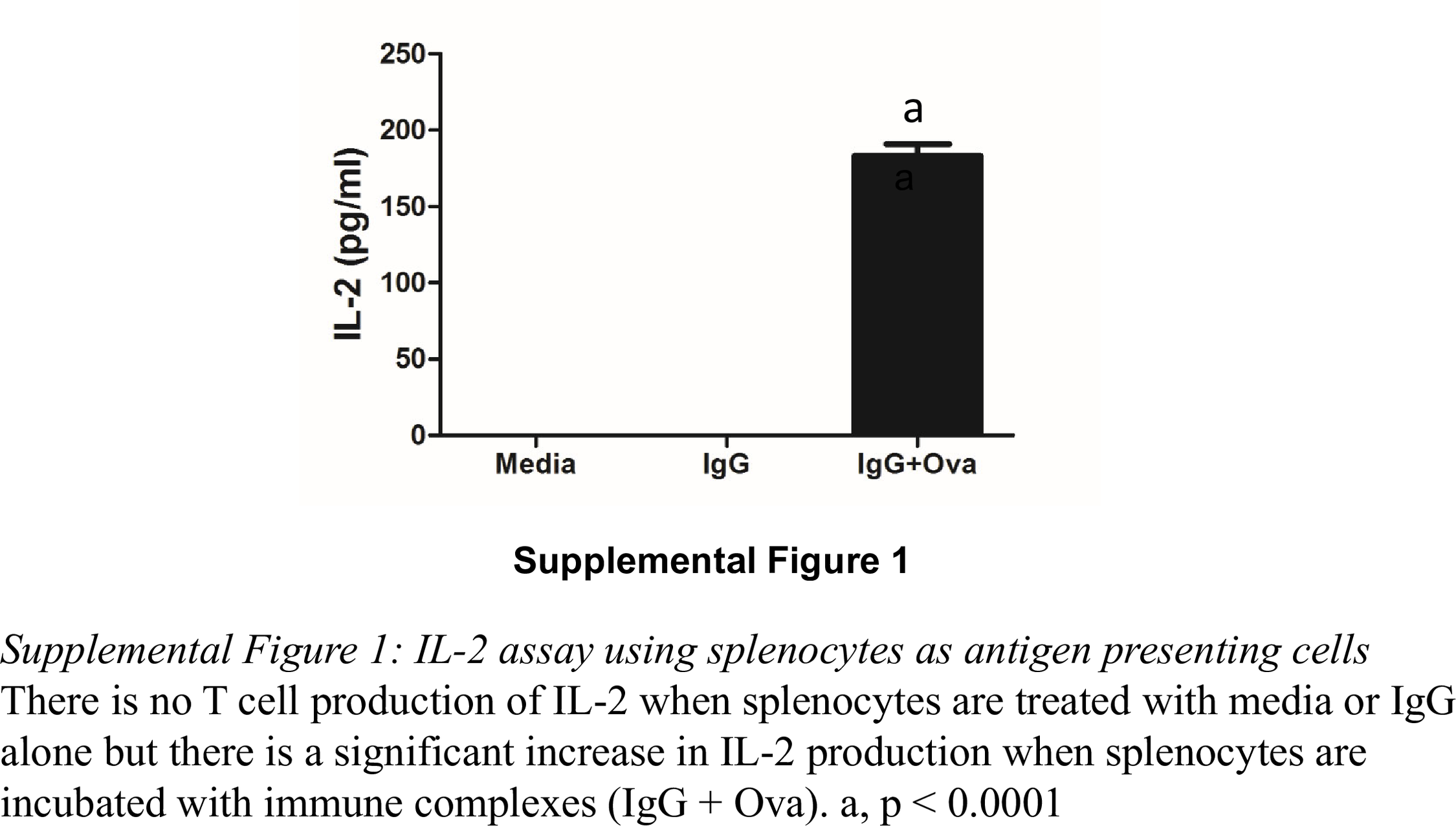

Supplement: S1 Fig — There is no T cell production of IL-2 when splenocytes are treated with media or IgG alone but there is a significant increase in IL-2 production when splenocytes are incubated wth immune complexes (IgG + Ova). a, p<0.001. (TIF) [file pone.0230401.s001.tif]
