# Supplementary material for: Podocyte-specific knockout of the neonatal Fc receptor (FcRn) results in differential protection depending on the model of glomerulonephritis
Source: PLoS One. 2020 Dec 28;15(12):e0230401. doi: 10.1371/journal.pone.0230401 (PMC7769425; doi:10.1371/journal.pone.0230401)
Supplement: S2 Fig — There was no difference in glomerulosclerosis scores between control mice and podocyte FcRn KO mice injected with saline (sal) nor with control IgG (IgG). n = 2 mice per group. Scale bars in lower left corner are 20μm. (PDF) [file pone.0230401.s002.pdf]

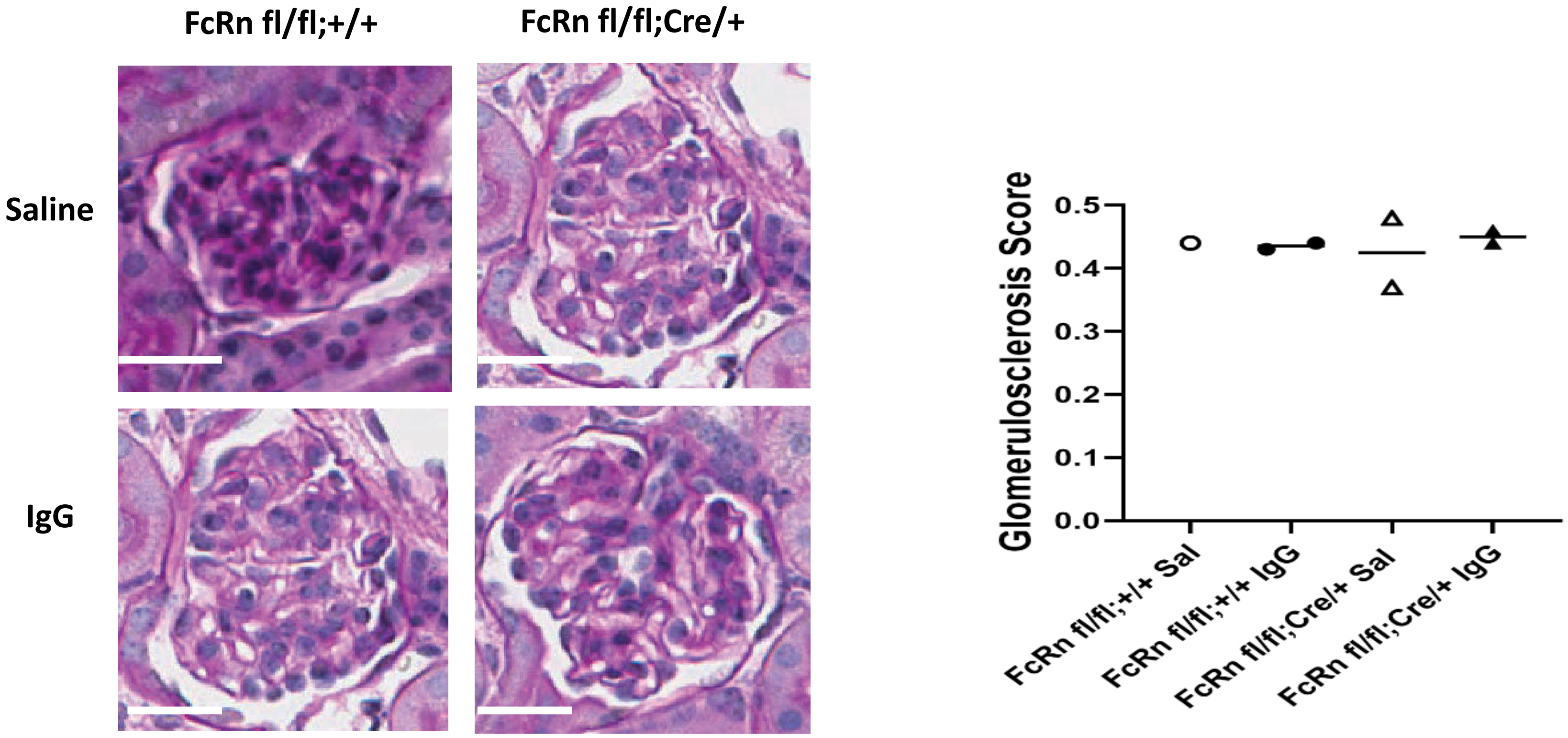

S2 Fig

S2 Fig: Histologic analysis of control and podocyte FcRn KO mice injected with IgG control and saline.
There was no difference in glomerulosclerosis scores between control mice and podocyte FcRn KO mice injected with saline (sal) nor with control IgG (IgG). n=2 mice per group. Scale bars in lower left corner are 20μm.
